# Summer undergraduate research: A new pipeline for pain clinical practice and research

**DOI:** 10.1186/s12909-016-0648-7

**Published:** 2016-05-04

**Authors:** Benedict J. Kolber, Jelena M. Janjic, John A. Pollock, Kevin J. Tidgewell

**Affiliations:** Department of Biological Sciences, Bayer School of Natural and Environmental Sciences, Duquesne University, 600 Forbes Avenue, Pittsburgh, PA 15219 USA; Graduate School of Pharmaceutical Sciences, Mylan School of Pharmacy, Duquesne University, 600 Forbes Avenue, Pittsburgh, PA 15219 USA; Chronic Pain Research Consortium, Duquesne University, 600 Forbes Avenue, Pittsburgh, PA 15219 USA

**Keywords:** Undergraduate education, Undergraduate medical education, Pain, Medical education

## Abstract

**Background:**

Most medical schools fail to provide adequate training of clinicians in the treatment of pain. Similarly, despite the fact that over 1/3 of Americans suffer from chronic pain, National Institutes of Health (NIH) funding for pain represents only ~1 % of the NIH budget. These issues may dissuade students from pursing pain in their clinical and research careers. To address these gaps in training and funding, we argue that exposing students to pain science early in their careers, at the undergraduate level, may be an effective method to develop a pipeline for future pain clinicians and scientists. To highlight our argument, we will describe our recent successful implementation of a cross-disciplinary and community-engaged biomedical summer research program. The Pain Undergraduate Research Experience (PURE) summer program involved both off-site and on-site experiences to expose undergraduate students to the range of careers in the pain field from basic science to clinical practice. The objective of the 10-week long PURE program was to evaluate whether a combination of basic science research, clinical practice visits, and patient interactions would increase student understanding of and exposure to the underlying science of pain.

**Methods:**

A pre-post cohort study was used without a comparison group. Entry and exit surveys were used to evaluate students’ perceptions about pain clinical practice and research, student interest in pain, and student confidence about communicating about pain and doing basic science pain research.

**Results:**

Students reported significant increases to a number of questions in the survey. Questions were scored on 5 point Likert scales and there was significant increases in student understanding of what life is like with chronic pain (2.6 vs 4.3 post survey), their confidence in explaining pain to a patient (2.8 vs 4.1) or researcher (2.8 vs 4), and their comfort with pain terminology(2.8 vs 3.9).

**Conclusions:**

With the PURE program, we wanted to entice top undergraduates to consider pain as a future area of study, practice, and/or research. We present a model that can be easily implemented at research universities throughout the United States.

## Background

The prevalence and cost of chronic pain in the United States is staggering. In the United States, the Institute of Medicine (IOM) estimated 5 years ago that over 100 million individuals suffer from chronic pain at a cost of $560–636 billion [1]. Half a decade late, we anticipate that the number of individuals affected and the personal an economic costs are even higher. Not surprisingly, pain is one of the main reasons that patients visit a primary care physician (PCP). Unfortunately, while the problem of chronic pain has been acknowledged, the resources necessary to properly tackle the problem and effectively treat patients have been elusive [2]. Although there are many impediments to pain therapy the most salient include the lack of adequate graduate-level training for those interested in clinical practice [3–5] and the disproportionate lack of funding for basic and clinical research in pain [6].

The notion that a focus on pain education in medicine (i.e. medical school, nursing, pharmacy, physical therapy, etc) is critical to a strong healthcare system is not a new idea. The first comprehensive suggestions for pain medical curriculum were developed in the 1980s [3] and have been updated numerous times to reflect current knowledge about the neurobiology and clinical outcomes of pain as well as new emphases on core competencies and interprofessional coordination [7]. Proper education on pain management, treatment, neurobiology, and patient empathy impacts patient outcomes and providers’ confidence in dealing with patients suffering from both acute and chronic pain conditions [4, 5]. Models of education that incorporate a comprehensive view-point on pain treatment including both specific neurobiological knowledge coupled with emotional development have been proposed as ideal for the treatment of pain where empathy for the patient can shape treatment outcomes [8]. Improved empathy for patients increases their ratings of interpersonal care and may help build the trust necessary for the long-term management of a chronic pain condition [9]. The majority of chronic pain patients are treated by PCPs [10]. PCPs tend to prescribe non-optimal amounts of opioids for the long-term management of chronic pain patients [10]. This behavior has been ascribed to concern about regulatory oversight and low confidence and knowledge in PCPs ability to treat neuropathic and musculoskeletal pain. Low confidence in treating pain is also correlated with the likelihood that a PCP will refer a patient to a pain specialist [10]. This association between non-optimal treatment and low confidence in treating chronic pain may be one symptom of a systemic failure in medical education to provide sufficient pain education.

Although early studies documenting the presence or absence of pain education in medical schools described rates as high as 84 % of schools having any amount of education on pain [11], there is a wide-spread recognition that pain education is insufficient in clinical education with regards to the depth of study and the lack of pain-specific courses [12]. Clearly schools with no pain education are far behind the need for pain education. However, there is evidence that even schools that do include pain education during didactic or clinical education may not be offering sufficient or comprehensive instruction on pain. Having a few hours of education in the context of 1000s of hours of total training is likely insufficient compared to curricula that include dedicated pain-specific courses during clinical education. Newer studies, including a comprehensive analysis of allopathic medical schools in the United States as well as Canada, have incorporated more detailed mapping of the types and amount of pain education that is occurring [12, 13]. As of 2011, 20 % of medical schools in the United States report no pain education and 20 % report having five or fewer hours of pain education [12]. Only 4 % of medical programs include comprehensive pain education including dedicated courses or mini-courses focused on pain [12]. Despite these disappointing numbers, there are a number of institutions that have embraced the idea of comprehensive pain education [14]. Such programs, including those funded through the National Institutes of Health’s (NIH) Pain Consortium Center of Excellence in Pain Education (CoEPE) [15], incorporate active learning approaches to pain education that focus on interprofessional methods of pain treatment and management. Nonetheless, the overall under representation of pain education coupled with increased public scrutiny of opioid treatment options may ultimately discourage talented up-and-coming students from focusing on pain. This sort of attitude negatively reinforces the lack of pain education and further prevents knowledge gained from basic and clinical research from moving quickly into the clinic. The low level of pain-related research funding available from federal agencies further hampers this knowledge flow.

As described above, the annual cost of chronic pain treatment and lost productivity is well over $500 billion. In stark contrast, the total amount of the NIH budget dedicated to pain research in 2014 was only $499 million [16]. Other federal agencies including the Department of Defense and Veteran’s Affairs also fund little research into pain, despite an obvious need from their constituents [2]. This underfunding of pain research means that clinicians do not have a complete picture of the long-term use of most pain therapies in distinct patient populations. This is particularly relevant in the context of long-term opioid use. Reduced research funding also limits the development of new therapies, especially from novel sources discovered through basic science research [2]. Finally, we reason that reduced funding drives stagnation in the pain research by limiting movement of new researchers into the field as is thought to be happening across biomedical research [6].

The solutions to the problems described are obviously complex and multi-fold. Significant buy-in from universities and the NIH will ultimately be necessary to improve patient outcomes through better clinical interactions and the development of better therapies. We can, however, work within the current system to ensure that there is a steady stream of new students and scientists that are not only interested in pain but also passionate about research and the application of effective therapy. To address these gaps in training and funding, we felt that exposing students to the science of pain and related research early in their careers may be an effective method to develop a pipeline for future pain scientists and clinicians. In this article, we will describe our recent successful implementation of a cross-disciplinary and community-engaged biomedical summer research program. The Pain Undergraduate Research Experience (PURE) summer program involved both off-site and on-site experiences and developed out of our Chronic Pain Research Consortium (CPRC) [17]. The CPRC was founded in 2011 by Janjic, who was a chronic pain patient at the time. Its mission is to enhance pain research and education and the PURE program embodies its core founding principles. The PURE program was built to entice well-qualified undergraduates to think about pain from an early stage of their careers. For those undergraduate research scholars who intend to seek a career in medicine, our solution develops groups of students that are in-tune to the need for pain education prior to medical school and will provide a remedy to the fragmented pain education that they may receive. The idea is that if a student understands the need for comprehensive pain training that they will be able to better integrate the fragmented pain education that they receive in medical school. Conversely, for undergraduate scholars who intend to seek a career in research, we want them to be targeting cutting-edge laboratories that focus on pain for their graduate education. Ultimately, whether students go into clinical practice or research, or other fields, our hope is that pain will be on their minds and they can be agents for change. The aim of this study was to evaluate whether a pain-specific undergraduate research program would improve undergraduate student’s understanding of and interest in pain clinical practice and research.

## Methods

### Design and setting of study

This study was done as a pre-post cohort study and was intended as a descriptive study to generate preliminary findings about the PURE program, possible barriers to its intended function, and initial level of pain knowledge in the population where future participants will come from. The design of the study was based on a typical summer undergraduate research experience with a novel focus on 10-weeks of intensive pain research. In addition, all of our PURE fellows participated in off-site and on-site experiences with community members. Off-site, working with two different pain clinics, individual students spent time shadowing clinicians as they interviewed, interacted, and treated chronic pain patients. Clinical shadowing experiences have been linked to increased empathy with the patient condition and increased interest in post-graduate studies [18]. On-site at Duquesne, students had a group session with a pain psychologist from the community and separately a fibromyalgia chronic pain patient. The goal for these sessions was for students to be able to understand pain from both the clinical and patient perspective in an informal and relaxed atmosphere. In addition to these community partnerships, the PURE program also involved a monthly pain journal club, weekly pain textbook seminar, a community service project, process-oriented training sessions (e.g. poster making, abstract writing), a 6-week ethics session, and individual presentations at a regional poster symposium. In addition, all of our students travelled off campus to attend a regional pain research conference. The goal for this experience was to provide students with exposure to some of the best pain research in the area and world. Setting of study was Duquesne University, a research university in Pittsburgh, PA, USA.

### Participants and participant selection

The study population were rising sophomores, juniors, and seniors from 4-year undergraduate universities and colleges in the United States. Participants applied for the PURE program through an online application process. Application materials included an academic transcript, two letters of recommendation, a resume, and a statement of goals. The applicants were asked to describe their specific interest in pain and their preferred research lab in the statement of goals. We received a total of 39 completed applications for the program including 30 external applications. Applicants were evaluated by a committee including individuals within the Duquesne pain community and outside. Applicants were based on a combination of factors including overall GPA, GPA in science courses, letters of recommendation, statement of goals, and availability of faculty mentors to host students.

### Consent and ethical considerations

After enrollment in the PURE program, participants gave oral and written informed consent. All 10 students enrolled in the 2015 PURE program consented to participate in the study. Consent document included the following sections: purpose of the study, compensation (none for participation in the study), confidentiality, right to withdraw, and a statement of voluntary consent. Voluntary consent statement was as follows—“I have read the above statements and understand what is being asked of me. I also understand that my participation is voluntary and that I am free to withdraw my consent at any time, for any reason. Withdrawal from the study will in no way affect the educational services I will receive as a summer researcher at Duquesne University. Finally, I affirm that I am at least 18 years old. On these terms, I certify that I am willing to participate in this research project.” Risks associated with participation in this study were deemed to be minimal. As described below, all surveys were coded without personal information. This study was determined to be “Exempt” according to “US 45CRF46.101(b)(2): Anonymous Surveys—No Risk.”

### Surveys

To evaluate student’s perceptions of undergraduate research, pain clinical experiences, pain research, and presentation of scientific data about pain. We used an entry and exit survey (Table 1) given during the first and last weeks of the 10-week program. Each survey took less than 30 min to complete and included demographic questions, qualitative free-answer questions, and Likert-type questions. Survey included similar content to the well established and validated SURE questionnaire [19] along with pain-specific questions. The SURE survey focuses on generic summer research programs with the goal of mapping out undergraduate interest in science careers. Our surveys contain modifications to suit our specific needs including: asking specific questions about pain and neurodegeneration, evaluating the impact of our clinical shadowing experiences, and evaluating student perceptions about their ability to communicate with other pain and neurodegeneration researchers. Surveys were blinded and coded for comparison of paired entry and exit surveys. No identifying information was included in the survey or coding.

**Table 1:** Entry and exit surveys

Entry Survey	Exit Survey
Survey	
Participant #	Participant #
Sex	
Highest Year Completed in College	
What is your current GPA?	Q1: Did you participate in the Duquesne PURE program?
Q2: On a scale of 1–5, how would you evaluate the quantity of academic interactions with your mentors at your home institution?	Q2: On a scale of 1–5, how would you evaluate the quantity of academic interactions with your mentors in the PURE program?
Q3: On a scale of 1–5, how would you evaluate the quality of these interactions at your home institution?	Q3: On a scale of 1–5, how would you evaluate the quality of these interactions in the PURE program?
Q4: On a scale of 1–5, to what extent did your home institution mentor encourage you to participate and help you find a summer research program?	Q4: On a scale of 1–5, to what extent did your PURE mentor help you set these goals?
Q5: On a scale of 1–5, how well do you feel your home institution has prepared you in terms of conceptual and theoretical knowledge?	Q5: On a scale of 1–5, how would you evaluate the quality of technical expertise, in terms of conceptual and theoretical knowledge, you gained from the program?
Q6: Indicate your agreement to the following statement: I have been introduced to the scientific method and/or participated in scientific research outside the classroom.	Q6: Indicate your agreement to the following statement: I have been introduced to the scientific method and/or participated in scientific research outside of the classroom.
Q7: On a sclae of 1–5, how would you evaluate your ability as a scientific speaker?	Q7: On a scale of 1–5, how would you evaluate your ability as a scientific speaker?
Q8: On a scale of 1–5, how would you evaluate your ability as a scientific writer?	Q8: On a scale of 1–5, how would you evaluate your ability as a scientific writer?
Q9: On a scale of 1–5, how important do you feel the skills in question 7 and 8 are to your professional development (i.e. do you think you will ever use them)?	Q9: On a scale of 1–5, how important do you feel the skills in question 7 and 8 are to your professional development (i.e. do you think you will ever use them)?
Q10: On a scale of 1–5, how would you evaluate your ability to interact with your peers?	Q10: On a scale of 1–5, how would you evaluate your ability to interact with your peers?
Q11: On a scale of 1–5, how would you evaluate your ability to perform scientific literature searches?	Q11: On a scale of 1–5, how would you evaluate your ability to perform scientific literature searches?
Q12: On a scale of 1–5, how would you evaluate your ability to prepare a scientific abstract?	Q12: On a scale of 1–5, how would you evaluate your ability to prepare a scientific abstract?
Q13: On a scale of 1–5, how would you evaluate your ability to prepare a scientific poster?	Q13: On a scale of 1–5, how would you evaluate your ability to prepare a scientific poster?
Q14: On a scale of 1–5, how would you rate your time management skills?	Q14: On a scale of 1–5, how would you rate your time management skills?
Q15: On a scale of 1–5, how would you evaluate your critical thinking ability?	Q15: On a scale of 1–5, how would you evaluate your critical thinking ability?
Q16: On a scale of 1–5, how would you evaluate the effectiveness of the PURE/URP application process?	Q16: On a scale of 1–5, how would you evaluate the effectiveness of the PURE/URP speakers/seminars?
Q17: On a scale of 1–5, how would you evaluate the pre-arrival contacts with faculty?	Q17: On a scale of 1 to 5, how would you evaluate the PURE off-site clinical experience visit?
Q18: On a scale of 1–5. how would you evaluate the arrival process and accomodations?	Q18: On a scale of 1 to 5, how would you rate the PURE on-sute interaction with the clinician?
Q19: On a scale of 1–5, how would you evaluate the PURE program webpage?	Q19: On a scale of 1 to 5, how would you evaluate the PURE on-site interaction with a chronic pain patient?
Q20: On a scale of 1–5, how well do you think you understand what it is like to live with chronic pain?	Q20: On a scale of 1 to 5, how would you evaluate learning about and building a pain “empathy” kit?
Q21: On a scale of 1–5, how likely are you to enter into clinical practice treating pain in the future?	Q21: On a scale of 1 to 5, how likely would you be to recommend the PURE program to a peer?
Q22: On a scale of 1–5, how likely are you to enter into basic science research to understand pain or chronic pain?	Q22: On a scale of 1 to 5, how well do you think you understand what it is like to live with chronic pain?
Q23: As of today, how comfortable do you feel researching pain and/or chronic pain that you have not encountered previously?	Q23: On a scale of 1 to 5, how likely are you to enter into clinical practice treating pain patients in the future?
Q24: AS of today, how comfortable do you feel explaining pain and/or chronic pain to another researcher?	Q24: On a scale of 1 to 5, how likely are you to enter into basic science research to understand pain or chronic pain?
Q25: As of today, how comfortable do you feel explaining pain and/or chronic pain to a patient in lay terms?	Q25: As of today, how comfortable do you feel researching pain and/or chronic pain that you have not encountered previously?
Q26: How comfortable are you with pain research terminology?	Q26: As of today, how comfortable do you feel explaining pain and/or chronic pain to another researcher?
Q27: How necessary is it to use animal models in pain research?	Q27: As of today, how comfortable do you feel explaining pain and/or chronic pain to a patient in lay terms?
Q28: Rank the following influences on your selection of the summer research experience at Duquesne University: Location, Academic Excellence, Specific Research Project, Specific Advisor, Curiosity in Pain Research.	Q28: How comfortable are you with pain research terminology?
Q29: What is your definition of pain?	Q29: How necessary is it to use animal models in pain research?
Q30: What are your career goals entering the PURE program (be honest)?	Q30: What is your definition of pain?
Q31: What are your expectations of the summer research program?	Q31: Has being involved in the PURE program changed or reinforced your career goals be honest)?
	Q32: Is there anything that could be done to improve the PURE program?

Questions given on the entry and exit surveys completed by all participants

### Statistical analyses

Evaluation of student responses for the “definition of pain” was done blinded to individual participant and whether the definition was from an entry or exit survey by two researchers. Rubric for scoring was developed *de novo* based on the International Association for the Study of Pain (IASP) taxonomy of pain definition “An actual sensory and emotional experience associated with actual or potential tissue damage, or described in terms of such damage” [20]. The maximum possible points on the rubric was 9. The highest value obtained from PURE fellows was 4.5. For the definition question, the score on the exit survey was divided by the entry survey score to determine a percent increase in score. This was analyzed by a 1-sample t-test to determine if students showed an increase or decrease in the accuracy of their definition. In addition, for the definition questions and other questions that were repeated on the entry and exit survey’s, student responses were evaluated using paired t-tests. Data are presented as mean ± SEM. All differences with a *P* < 0.05 were considered statistically significant.

## Results

**Fig. 1:**
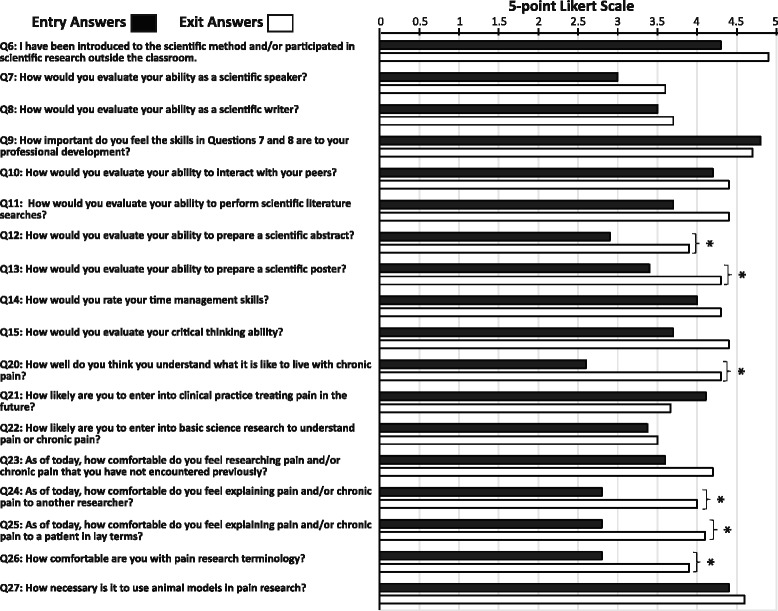
Summary of Entry and Exit Surveys. Students were given entry and exit surveys to evaluate their career motivations, comfort describing pain to patients and researchers, familiarity with pain terminology, and confidence presenting and understanding scientific data. Data were collected using surveys with 5-point Likert-type scales. Data shown are for questions with both entry and exit answers only. Values for answers to entry surveys are represented by black bars and answers to exit surveys are represented by white bars. Increases were seen for nearly all statements or questions presented to the students comparing the exit surveys to the entry surveys. An exception to this increase was the small decrease (not significant) in the likelihood for students to be pain clinicians in the future. This decrease was accompanied by a small increase (not significant) in the likelihood that they would pursue a research career instead. There were statistically significant differences (paired t-test *P* < 0.05) in students’ self-reported perceptions of the following questions: Question 12 - How would you evaluate your ability to create a scientific abstract (scale “very weak” to “very strong”; *p* = 0.015). Question 13 - How would you evaluate your ability to create a scientific poster (scale “very weak” to “very strong”; *p* = 0.041). Question 20 - How well do you think you understand what it is like to live with chronic pain (scale “not well” to “very well” *p* = 0.002). Question 24 - How comfortable do you feel explaining pain to another researcher (scale “very uncomfortable” to “very comfortable”; *p* = 0.00096). Question 25 - How comfortable do you feel explaining pain to a patient in lay terms (scale “very uncomfortable” to “very comfortable”; *p* = 0.017). Question 26 - How comfortable are you with pain research terminology (scale “very uncomfortable” to “very comfortable”; *p* = 0.0067)

Student attitudes, comfort, and knowledge about pain were assessed using entry and exit surveys (Table 1). These surveys were used to measure insight into what the summer experience was like and how students’ perceptions of pain and pain research changed during the summer. All collected data was blinded and the study was approved by the Duquesne University Institutional Review Board for Human Studies. For all measures, increases or relevant decreases were seen including a number of measures that showed significant differences between entry and exit surveys (Fig. 1). Descriptions of each activity follow along with outcomes.

### Demographics

Students (*n* = 10; eight female; all GPA >2.5; variety of years in college) came from Duquesne University (*n* = 8) or two external “liberal arts” institutions. Duquesne students came from three different schools on campus (science, health science, pharmacy) indicative of the multi-disciplinary nature of pain research. All students reported strong qualitative support from their home institutions and previous research and academic mentors on entry surveys. To the question “On a scale of 1–5, how would you evaluate the quantity of academic interactions with your mentors at your home institution?” the average student response was 4.4 ± 0.2. To the question “On a scale of 1–5, how would you evaluate the quality of these interactions at your home institution?” the average student response was 4.4 ± 0.2. To the questions “On a scale of 1–5, to what extent did your home institution mentor encourage you to participate and help you find a summer research program?” the average student response was 4.2 ± 0.3. To the question “On a scale of 1–5, how well do you feel your home institution has prepared you in terms of conceptual and theoretical knowledge?” the average student response was 4.3 ± 0.3.

In addition, interactions with faculty members in the PURE were reported strongly positive and extensive on exit surveys. To the question “On a scale of 1–5, how would you evaluate the quantity of academic interactions with your mentors in the PURE program?” the average student response was 4.6 ± 0.2. To the question “On a scale of 1–5, how would you evaluate the quality of these interactions in the PURE program?” the average student response was 4.9 ± 0.1. To the question “On a scale of 1–5, to what extent did your PURE mentor help you set these goals?” the average student response was 4.5 ± 0.2. Students reported quality gains in technical knowledge related to pain. To the question “On a scale of 1–5, how would you evaluate the quality of technical expertise, in terms of conceptual and theoretical knowledge, you gained from the program?” the average student response was 4.6 ± 0.2.

### Pain research

Students were placed in research labs working on some aspect of pain. All faculty involved were part of the Duquesne CPRC. This consortium includes faculty involved in basic science pain research (pain biology and pharmacology, pharmaceutics, medicinal chemistry, natural products chemistry, nanomedicine, molecular imaging, pain in aging), pain clinical practice, and pain education. In combination with all of the clinical exposure from the planned activities, students were exposed to basic science research into mechanisms of pain, how to treat pain with mechanical interventions, analgesic drug discovery, pain education research, nanoemulsions for pain drug delivery, and understanding pain circuitry from a biological perspective.

### Pain journal club and didactic pain seminar

A solid understanding of primary literature and the current state of the field is important for young researchers. The journal club was presented by graduate students and faculty involved in pain research. Articles were chosen from a variety of fields and perspectives and sent to students 1 week before presentations. Presenters gave a brief background on the general topic and then broke down the paper and went through its main assumptions and results. Students were encouraged and asked to provide their input on these topics as well. This allowed students to be exposed to how literature can be evaluated and critically assessed. Sessions would typically end with a discussion about what the next steps in the research should be or how the paper might alter or affect their own research. In addition to this monthly journal club, PURE students were also encouraged to attend a didactic pain course run by PhD graduate students. In this informal course, a single graduate student went through chapters of Wall and Melzak’s The Textbook of Pain [21] each week. Consistent with expectations of learning from the journal club and course, didactic training significant improvements were seen in student’s “comfort with pain terminology” comparing entry and exit surveys (Fig. 1). Although students’ comfort with pain terminology increased during the summer, their ability to define pain did not significantly change (entry 2.3 ± 0.3 vs exit survey 2.8 ± 0.3 out of possible 9, paired t-test, *P* = 0.34; exit/entry score 142 ± 21 %, one-sample t-test compared to hypothetical “no change” value of 100 %, *P* = 0.08). Discussions during the journal club and in students’ research labs also likely contributed to increases in students’ “comfort…explaining pain and/or chronic pain to another researcher” (Fig. 1). To the question “On a scale of 1–5, how would you evaluate the effectiveness of the PURE/URP speakers/seminars?” the average student response was 4.5 ± 0.2.

### Interaction with the clinical side of pain

In designing the PURE program, we wanted students to appreciate why they were doing basic science research. While part of the answer to this question lies in the specific research project that each student worked on, the bigger answer focuses primarily on the needs and experiences of individuals living with pain. To develop the skill-set and empathy necessary to interact with patients and other clinicians, students participated in three “clinical” activities. They shadowed pain doctors at a pain clinic, met informally at Duquesne with a pain patient, and had lunch with a pain psychologist.

For the clinical shadowing component of the program, students went individually or in pairs to one of two pain clinics in Pittsburgh, PA. Students spent ~4 h at the pain clinic where they observed doctor-patient interactions and pain intervention procedures. Both clinicians reported these events as positive experiences; “The students were eager, engaging, and interacted well with staff and patients” (e-mail conversation between BJK and clinicians). To the question “On a scale of 1 to 5, how would you evaluate the PURE off-site clinical experience visit?” the average student response was 4.9 ± 0.1.

For the on-campus visit with a pain patient, students were able to interact informally with a long-term (>15 year) fibromyalgia patient. This patient had been briefed prior to the session to establish appropriate boundaries and expectations. These boundaries were then shared with the students prior to the start of the session. During this informal 1-hour session, the patient described her experience living with chronic pain and her experiences interacting with various pain clinicians. The students were encouraged (by the patient) to then ask questions. One of the advantages of this situation compared to patient interactions in a formal clinic is the freedom that the students felt. They were able to ask “stupid” questions that would be deemed inappropriate in a clinical setting. The patient described the experience as positive and was encouraged to see so many young scientists working in pain basic science research (personal correspondence between BJK and patient). To the question “On a scale of 1 to 5, how would you evaluate the PURE on-site interaction with a chronic pain patient?” the average student response was 4.5 ± 0.3.

Finally, on-campus at Duquesne, students also had the opportunity to have an informal lunch with a pain psychologist. This clinician described their reasons for being a pain specialist as well as the day-to-day activities involved in their clinical practice. The goal of this session was for students to once again be able to ask “prying” questions about life as a clinician. To the question “On a scale of 1 to 5, how would you rate the PURE on-site interaction with the clinician?” the average student response was 4.5 ± 0.2.

Overall, the patient and clinical interactions were seen as universally positive by students, clinicians, and the on-site patient. In exit surveys students reported significant increases in their ability to “understand what it is like to live with chronic pain” and to “explain pain and/or chronic pain to a patient in lay terms” (Fig. 1).

### Pain empathy kit

At Duquesne University, two members of the CPRC, Diane Rhodes and Dr. Lynn Simko, run a semester-long interprofessional course (Pharmacy and Nursing students) dedicated to pain. As part of the course, these researchers developed a pain “empathy kit” that allows people to empathize with chronic pain patients by altering their abilities and sensations to match those of a pain patient. In the PURE program, students were tasked with putting together and using the empathy kit as well as trying to think of new simulations or activities to add to the kit. When using the kit with groups, the session began with a brief overview of pain assessment and etiology, which was then followed by the use of activities to simulate life with chronic pain. The activities were designed to not cause personal harm to users. Examples of activities were walking around the room with popcorn kernels in the bottoms of one’s shoes to simulate peripheral neuropathy or wearing a belt with a golf ball attached at the lower back to simulate chronic low back pain. This kit can easily be assembled and utilized by other programs and courses in order to not only teach and demonstrate physical detriments caused by chronic pain but also to increase empathy and emotional understanding of the daily struggle of pain patients. Improvement of empathy with patients has been shown to improve patient outcomes in the clinic [9]. To the question “On a scale of 1 to 5, how would you evaluate learning and building a pain empathy kit?” the average student response was 4.1 ± 0.3.

### Ethics program

All students participated in a 6-week long ethics program that is used by Duquesne’s non-discipline specific Undergraduate Research Program. This ethics program involves groups of students tackling published scientific and medical ethics problems. Students identify the problem, the events that led to the ethical lapse, and methods to avoid similar mistakes in the future. Each group of students is mentored by a faculty member or graduate student during the 6-week process.

### Pain conference

Finally, to again expose students to a diversity of pain research topics and to improve their ability to discuss their own research, students participated in two additional activities. After 5 weeks, students spent 2 days at the University of Pittsburgh attending a pain-research conference (http://paincenter.pitt.edu/barriers.htm). Although this conference was a singular event, the incorporation of additional exposure to world-class pain research allowed the students to appreciate the diversity of open questions with the pain research field. The final activity of the summer involved students presenting the results of their research at a 1-day regional scientific conference at Duquesne to a broad audience of researchers. Their research was presented in the form of scientific posters. As a group, the students were given a lesson in poster layout and presentation style and practiced draft versions of their posters prior to the research conference. Students reported significant gains in their ability to “prepare a scientific abstract” and to “prepare a scientific poster” (Fig. 1). Several students have gone on to present their research at additional regional or international conferences after the summer program concluded.

## Discussion

The core of the PURE program was an intensive full-time pain-specific undergraduate research program. Students applied for and were selected to work in different research labs during summer 2015 for 10 weeks. As is well established, intensive summer research experiences increase scientific literacy overall and increase the chance that a student focuses on research in their career [22, 23]. However, we also recognized that simply having students work in a pain research laboratory would not give students the comprehensive and multi-disciplinary experience that is needed for individuals working in the pain field. So, we partnered with members of the Pittsburgh community to offer a more complete and authentic experience. Each of these experiences was designed to enhance the students’ understanding of chronic pain and/or expose them to areas of pain clinical practice and research. Overall, students experienced gains in their familiarity with pain terminology, comfort communicating about pain to patients and researchers, and their ability to present scientific data.

Exposure to pain research is not something that students typically receive during their undergraduate time. The PURE program had a positive impact on students understanding of pain and pain research and helped educate them on the diversity of research and work being conducted. Hands-on research provides motivation students to stay in STEM fields [24]. The PURE program likely enhances this effect through the clinical exposure that strengthens the student’s connection to their research. The focus on pain for the PURE program was driven by data showing increasing rates of pain [1] and poor post-graduate training in pain [12]. A analogous approach was used in the Summer Training in Aging Research Topics—In Mental Health (START-MH) program funded by the National Institute of Mental Health to encourage students to enter the field of geriatric psychiatry [25]. This program found that the model could be used to build a pipeline for geriatric medicine. An important difference between the PURE and the START-MH is that all students in the START-MH trained at different institutions in the United States. Having all of our PURE fellows at one institution allowed us to develop activities to improve student’s professional development (e.g. seminar on poster presentations) and deepen their clinical exposure (e.g. informal lunches with patients).

Most of the students entering into the PURE program stated that they were highly likely to pursue a clinical career. Reinforcing this desire, at the end of the summer, in open-ended comments, students asked for more clinical shadowing experiences in future versions of the PURE. They also describe a small shift to think about research as an option including listing “MD/PhD” as a career option compared to “MD” only at the start of the summer. This new insight for the students into the research side of clinical practice may help focus the students’ behaviors in their future careers. We do not feel that this small shift in thinking about research instead of the clinic is representative of discomfort about pursuing a career in medicine. Instead, it likely reflects the students’ new and more diverse experience in the field of pain (i.e. both clinical and research experience). This diversity will likely help create medical students and professionals that are better equipped to read and address primary literature in their practice and future research experiences. In addition, evidence suggests that research experiences during medical school increase students’ productivity and participation in clinically-driven research in the future [26]. There is no reason to suggest that similar effects would not occur in our PURE fellows. Our plans for long-term follow-up of these students will help measure these long-term outcomes.

There are some limitations of this study that should be taken into account. These include the pre-post cohort study design, which prevented the comparison of the intervention (i.e. an intensive pain summer experience) to a control intervention. While such a comparison would be valuable for analytic purposes it would not be feasible from a financial perspective and might lead to students in a control group (e.g. summer research with no clinical component) being at a disadvantage in their professional development. The current study identified that students do have limited barriers to define pain and communicate about pain to peers and patients. Going forward with this program we will attempt to identify these barriers to enhance future offerings and advance pain education. A final limitation of this study was the fairly low number of participants in the PURE program. The number of subjects was ultimately limited by the cost of the summer research program (+$8000 per participant). Nonetheless, despite these limitations we do feel that this study shows the potential of intensive and focused summer undergraduate programs in the construction of biomedical pipelines for pain and other areas of education need.

The Affordable Care Act singles out treatment of chronic disease has a critical aspect in the future of the health care system in the United States [2]. As a field we need to be poised to respond to this challenge by providing opportunities for motivated students to specialize and understand both the research and clinical challenges associated with chronic disease.

Importantly, we feel that similar pain-specific undergraduate-directed programs can be easily created. At large research institutions with sufficient pain research, there is a ready pool of qualified research mentors and likely highly qualified undergraduate candidates. Using existing clinical contacts, it would be relatively straightforward to incorporate clinical shadowing opportunities as well. Of note, at least one other institution, the University of Washington, also has a similar program, the “Innovations in Pain Research Summer Program [27]” although no published data is available from this program. The effort and costs involved in developing a program such as the PURE can be mitigated by incorporating clinically driven summer research programs into an institution‘s existing basic science summer research program. We utilized this strategy to provide our 10 PURE fellows with opportunities to engage on a social and scientific level with the larger community of undergraduate researchers (~70) who participated in Duquesne University’s Undergraduate Research Program (URP). We were also able to use the existing application structure of the URP to attract candidates for the PURE. We received 39 applications including 30 external applications despite the fact that we did not explicitly advertise for the program.

## Conclusions

Broad incorporation of undergraduate student researchers into biomedical research laboratories including pain basic science laboratories with clinical exposure will help ensure that the next generation of pain clinicians and basic scientists is passionate about treating pain before even entering post-graduate education.

### Ethics approval and consent to participate

Study was approved by the Duquesne University Institutional Review Board (protocol #2015-05-10).

### Consent for publication

All data from participants was blinded and written consent was given for all participant data presented.

### Data availability

Raw data is available for download on the website http://www.duq.edu/about/centers-and-institutes/chronic-pain-research-consortium/publications

## Abbreviations

CPRC: Chronic Pain Research Consortium
IASP: International Association for the Study of Pain
PCP: Primary Care Physician
PURE: Pain Undergraduate Research Program
